# Novel histone deacetylase inhibitors in clinical trials as anti-cancer agents

**DOI:** 10.1186/1756-8722-3-5

**Published:** 2010-02-04

**Authors:** Jiahuai Tan, Shundong Cang, Yuehua Ma, Richard L Petrillo, Delong Liu

**Affiliations:** 1 Department of Medicine, The Mount Vernon Hospital, Mount Vernon, NY, 10550, USA; 2 Department of Oncology, Henan Province People's Hospital, Zhengzhou, China; 3 Division of Oncology/Hematology, New York Medical College, Valhalla, NY 10595, USA

## Abstract

Histone deacetylases (HDACs) can regulate expression of tumor suppressor genes and activities of transcriptional factors involved in both cancer initiation and progression through alteration of either DNA or the structural components of chromatin. Recently, the role of gene repression through modulation such as acetylation in cancer patients has been clinically validated with several inhibitors of HDACs. One of the HDAC inhibitors, vorinostat, has been approved by FDA for treating cutaneous T-cell lymphoma (CTCL) for patients with progressive, persistent, or recurrent disease on or following two systemic therapies. Other inhibitors, for example, FK228, PXD101, PCI-24781, ITF2357, MGCD0103, MS-275, valproic acid and LBH589 have also demonstrated therapeutic potential as monotherapy or combination with other anti-tumor drugs in CTCL and other malignancies. At least 80 clinical trials are underway, testing more than eleven different HDAC inhibitory agents including both hematological and solid malignancies. This review focuses on recent development in clinical trials testing HDAC inhibitors as anti-tumor agents.

## Background

Histones are among the most evolutionarily conserved proteins and the most abundant proteins bound to DNA in eukaryotic cells [1]. There are a total of five classes of them (H1, H2A, H2B, H3, and H4) organized into two groups: core histones (H2A, H2B, H3 and H4) and linker histone (H1). Two each of the core histones form nucleosome particle by wrapping 147 base pairs of DNA. Histone H1, as a linker, binds nucleosomes together and thus participates in a higher-order of histones as chromatin [2-4]. Chromatin undergoes modifications by changing its structure and chemical composition as cells differentiate, subsequently lead to diverse patterns of gene expression and differences in cellular function [5]. Such post-translational modifications are called epigenetic processes and are inheritable changes in gene expression without alteration of the nucleotide sequence [6]. These modifications in the chromatin including genomic DNA and histones or other chromatin-associated proteins comprise the addition of methyl, acetyl, and phosphoryl groups or even larger moieties such as binding of ubiquitin or small ubiquitin-like modifier [7,8]. Out of all the modifications above, histone acetylation is the most widely studied and has been shown to have diverse roles in the regulation of the nucleosome. Lysine acetylation, for example, can lead to changes in chromatin structure and may decrease the histone-DNA interaction and promote accessibility of the DNA for transcription activation [9]. The abnormal activation and deactivation of transcription based on histone acetylation status may be associated with tumorigenesis [10]. Several lines of evidence indicated that HDACs are associated with a number of well-characterized cellular oncogenes and tumor suppressor genes leading to development of many specific forms of malignancy [11,12].

In the eukaryotic cells, 18 different HDACs are identified and they may reside either in the nucleus or in the cytoplasm [13,14]. According to phylogenetic analyses and sequence homologies with yeast proteins, HDACs can be divided into four classes. Class I family of HDACs consists of 1, 2, 3 and 8 proteins. They are similar to yeast HDACs and locate in the nucleus of the cells exclusively [15,16]. Class II family members include 4, 5, 6, 7, 9 and 10, which are related to Hos3 in yeast. They primarily localize in the cytoplasm, but can transfer to nucleus from cytoplasm [17,18]. Class I and II of HDACs are evolutionarily related and share a common enzymatic mechanism, the Zn-catalyzed hydrolysis of the acetyl-lysine amide bond [19]. HDAC11 is located in both cytoplasm and nucleus and belongs to class IV [20]. It has a conserved domain in the catalytic region and shares features with both class I and II. The class III of HDACs is the so-called Sirts, consisting of seven members. These proteins are similar to Sirts in yeast. They are different with previous groups and are Zn-independent and dependent on NAD as a cofactor [21].

Inhibitors of HDACs were found to have anti-cancer function as a novel therapeutic class of drugs in many different cancers [22-26]. Based on their chemical structure, these inhibitors can be subdivided into four different classes, including hydroxamates, cyclic peptides, aliphatic acids and benzamides [27]. TSA, a compound of hydroxamates, is the first nature product that has been discovered to possess the HDAC inhibitor activity in1990. Its structural analog, suberoyl anilide hydroxamic acid (SAHA) was the first approved HDAC inhibitor for clinical treatment of T cell lymphoma. Other compounds, for example, CBHA [28,29] and LBH589 [30-32], have been used in pre- and clinical trials in this group. Another class of HDAC inhibitors is aliphatic acid, including Valproic acid (VPA) [33-35], phenylbutyrate [36]. The third group is benzamide consisted of MS-275 [25,37-41] and MGCD0103 [42-45]. The last group is cyclic peptide including FK-228 [46-50].

Although not fully understood, the clinical activity of these inhibitors is thought to be mediated in part by induction of histone acetylation, resulting in a permissive or more open chromatin configuration and potential reactivation of aberrantly suppressed genes resulting in growth arrest, cell differentiation, and apoptosis of tumor cells [51-55]. The patterns of alterations of gene expression are similar for different HDAC inhibitors, but show definite differences induced by different agents in various transformed cells [56-58]. Functionally, HDACs regulate gene expression by at least three mechanisms [59]. First of all, histone deacetylation increases the charge density on the N-termini of the core histones, thereby strengthening histone tail-DNA interactions and blocking access of the transcriptional machinery to the DNA template. In addition, histone modifications are specifically recognized by chromatin-interacting proteins [14]. A consequence of this alteration in nucleosome conformation is reduced accessibility of the transcriptional regulatory machinery to the DNA template, resulting in transcriptional repression [60-63]. A second mechanism by which HDACs regulate transcription is by catalyzing the deacetylation of sequence-specific DNA binding transcription factors. Acetylation and deacetylation of sequence-specific transcription factors can either increase or decrease their DNA binding activity, and subsequently may enhance or repress transcription [64-68]. Furthermore, a number of cytoplasmic proteins, including tubulin and HSP90, have now been shown to be acetylated by HDAC [69-73]

One of the HDAC inhibitors, vorinostat, has been approved by FDA for treating cutaneous T-cell lymphoma (CTCL) for patients with progressive, persistent, or recurrent disease on or following two systemic therapies. Other inhibitors, for example, FK228, PXD101, PCI-24781, ITF2357, MGCD0103, MS-275, valproic acid and LBH589 have also demonstrated therapeutic potential as monotherapy or combination with other anti-tumor drugs in CTCL and other malignancies. At least 80 clinical trials are underway, testing more than eleven different HDAC inhibitory agents including both hematological and solid malignancies. Vorinostat clinical trials have been updated lately [13,74]. This review focuses on recent development in clinical trials testing newer HDAC inhibitors as anti-tumor agents.

### PCI-24781

PCI-24781 (formerly CRA-024781) is a broad-spectrum phenyl hydroxamic acid. It has been evaluated alone or with ionizing radiation and other DNA-damaging agents in pre-clinical studies [75]. Recent pre-clinical data have suggested that it may act, in part, by inhibiting DNA repair resulted in a synergistic effect on apoptosis when combined with other agents [76,77]. Phase I clinical trial in refractory advanced solid tumor patients showed that PCI-24781 was well tolerated following intravenous or oral administration. Adverse events included anemia, thrombocytopenia, diarrhea, nausea, fatigue, and vomiting. Only one patient in the final cohort had asymptomatic non-specific ST- T wave changes and had drug discontinued. These were not dose-related. Mean oral bioavailability was 0.28 (34%) with no difference between solution and capsule. Tubulin and histone acetylation were documented in peripheral blood mononuclear cells (PBMCs). Acetylation levels increased at 1.5 h post dose and were sustained through 4 h in all patients. Stable disease up to 8 cycles was seen in 5 of 13 evaluable patients [78].

### ITF2357

ITF2357 is a synthesized HDAC inhibitor containing a hydroxamic acid moiety linked to an aromatic ring. Many reports demonstrated that it has inhibitory activity in the production of pro-inflammatory cytokines, as well as cytotoxic activity both in vitro on several human tumor cell lines and in vivo in patients with hematologic malignancies [79-83]. A phase II open label non randomized study was done at the National Tumor Institute of Milan using the drug as third-line or higher treatment of heavily pretreated, relapsed or refractory, Hodgkin lymphoma (HL) patients. Toxicity included: grade 1 leukopenia in 30%, grade 2 thrombocytopenia in 33%, fatigue in 50%, grade 1 diarrhea and/or abdominal pain in 40%; prolongation of QTc interval prompting transient drug discontinuation in 20%. Thirteen patients completed at least one cycle of therapy and were evaluated for response. Seven patients (54%) had stable disease by CT scan that was associated with a significant reduction in FDG-PET uptake in 6 patients (46%) lasting a median of 3 months. Six patients had progression of disease (POD). Preliminary results in this series of very heavily pretreated HL patients showed that oral ITF 2357 has anti-tumor activity and a good safety profile. The drug warrants additional studies, alone and in combination, as salvage treatment for HL even with less advanced disease [84].

### MS-275 (SNDX-275)

MS-275 is a synthetic benzamide derivative that has been shown to inhibit HDACs, and has anti-tumor activity in many preclinical models [25,85-88]. Clinical trial with this agent was first done in the patients with advanced solid tumors or lymphoma in 2005 (Table 1). They were treated with MS-275 orally initially on a once daily × 28 every 6 weeks schedule. The starting dose was 2 mg/m^2 ^and the dose was escalated in three- to six-patient cohorts based on toxicity assessments. With the daily schedule, the maximum tolerated dose (MTD) was exceeded at the first dose level. Therefore, once every 14 days schedule was implemented and found reasonably well tolerated. The MTD was 10 mg/m^2 ^and dose-limiting toxicities (DLTs) were nausea, vomiting, anorexia, and fatigue. HDAC inhibition was observed in PBMCs. Preliminary pharmacokinetics (PK) analysis suggested the half-life of MS-275 in humans was 39 to 80 hours, substantially longer than predicted by preclinical studies. Based on PK data, a more frequent dosing schedule, weekly × 4, repeated every 6 weeks is being evaluated [89]. A total of 22 patients were enrolled on this schedule, and 19 were considered evaluable for toxicity. The MTD was 6 mg/m^2^. No grade 4 toxicities were observed. DLTs were reversible and consisted of hypophosphatemia, hyponatremia, and hypoalbuminemia. MS-275 was found to be well tolerated at a dose of 6 mg/m^2 ^administered weekly with food for 4 weeks cycled every 6 weeks [90].

**Table 1:** Clinical studies of MS-275 (SNDX-275)

Phase	Other agent	Disease (pt. No.)	Schedule	Recommended dose	Reference
I		Relapsed or refractory AML (39).	Once weekly for 4 weeks of a 6 week cycle	8 mg/m^2^	[92]
I		Refractory solid tumors and lymphoid(22)	Once weekly for 4 weeks of a 6 week cycle	6 mg/m^2^	[90]
I		Refractory solid tumors and lymphoid(27)	Once weekly for 3 weeks of a 4 week cycle or once every other week.	4 mg/m^2^	[91]
I		Refractory solid tumors and lymphoid	Once every 2 week of 6 week cycle.	10 mg/m^2^	[89]

Three additional dose schedules were also studied: once every other week, twice weekly for 3 weeks every 28 days, and once weekly for 3 weeks every 28 days. MS-275 was confirmed to be safe and well tolerated at doses up to 6 mg/m^2 ^every other week or 4 mg/m^2 ^weekly for 3 weeks followed by 1 week of rest. These two schedules resulted in biologically relevant plasma concentrations and anti-tumor activity. Levels of histone H3 and H4 acetylation in PBMCs increased. Two of 27 patients showed partial remissions (PR), including one patient with metastatic melanoma who had a PR and has remained on study for >5 years. Six patients showed prolonged disease stabilization (SD). Twice-weekly dosing was not tolerable due to asthenia, and further evaluation of this schedule was halted. The recommended dose for further disease-focused studies is 4 mg/m^2 ^given weekly for 3 weeks every 28 days or 2 to 6 mg/m^2 ^given once every other week [91].

Phase 1 study in advanced acute leukemias also demonstrated that MS-275 was safe and can be tolerated at doses up to 8 mg/m^2 ^weekly for 4 weeks every 6 weeks. The patients were treated with MS-275 initially once weekly × 2, repeated every 4 weeks from 4 to 8 mg/m^2^, and after 13 patients were treated, once weekly × 4, repeated every 6 weeks from 8 to 10 mg/m^2^. DLTs included infections and neurologic toxicity manifesting as unsteady gait and somnolence. Other frequent non-DLTs were fatigue, anorexia, nausea, vomiting, hypoalbuminemia, and hypocalcaemia. Histone H3/H4 acetylation, p21 expression, and caspase-3 activation can be induced by MS-275 in bone marrow mononuclear cells. Even though MS-275 effectively inhibits HDAC in vivo in patients with advanced myeloid leukemias, responses by classical criteria were not seen [92].

Pre-clinical studies suggested that combining inhibitors of DNA methyltransferase (DNMT), 5-azacitidine (AZA), with inhibitors of HDAC, SNDX-275, synergistically induced re-expression of epigenetically-silenced tumor suppressor genes and had anti-tumor effect. Clinical study revealed it safe and well tolerated in 10 patients with advanced non small cell lung carcinoma (NSCLC). AZA was given subcutaneously on days 1-6 and 8-10 with SNDX-275 (MS-275) at a fixed dose of 7 mg/day on days 3 and 10 of a 28 day cycle. No DLT was seen in the 30 mg/m^2 ^dose cohort. At 40 mg/m^2^, one subject was replaced due to rapidly progressive disease during week 1. One subject experienced a hematologic DLT (grade 3 neutropenia and thrombocytopenia). No long term adverse outcomes from the DLT were seen. Common low grade toxicities included injection site reactions, nausea/vomiting, constipation, fatigue, and cytopenias. A major and durable PR has been observed in one patient, which is ongoing at >8 months. Two patients had stable diseases through ≥2 cycles of therapy; the remaining patients had PODs. This clinical trial showed that AZA and SNDX-275 combination may have clinical activity in advanced NSCLC patients after failing at least one previous chemotherapy regimen [93].

### Depsipeptide (romidepsin, FK228, FR901228)

Depsipeptide (FR901228) is a bicyclic peptide isolated from Chromobacterium Violaceum and has demonstrated potent in vitro cytotoxic activity against human tumor cell lines and in vivo efficacy against human tumor xenografts. Sander et al first studied 37 patients with advanced or refractory neoplasm by utilizing depsipeptide by a 4-h intravenous infusion on days 1 and 5 of a 21-day cycle in 2002 (Table 2). DLT included grade-3 fatigue (3 patients), grade-3 nausea and vomiting (1 patient), grade-4 thrombocytopenia (2 patients), and grade-4 cardiac arrhythmia (1 patient, atrial fibrillation). Reversible ECG changes with ST/T wave flattening were regularly observed. There were no clinically significant changes in left ventricular ejection fraction. The recommended Phase II dose is 17.8 mg/m^2 ^administered on day 1 and 5 of a 21-day cycle. One patient obtained a PR [94]. Other clinical study done in the similar population confirmed that depsipeptide can be safely administered when given as a 4-hour infusion and further clinical trials are warranted [95].

**Table 2:** Clinical studies of romidepsin (depsipeptide)

Phase	Other agent	Disease (pt. No.)	Schedule	Recommended dose & response	Reference
I		Advanced or refractory colorectal(11), renal (12)and other neoplasms(14)	Day1 and 5 of a 21-day cycle	24.9 mg/m^2^	[94]
I		Colorectal(8), breast(4), sarcoma(3) and other (15)	Day1, 8, and 15 of 28-day cycle	13.3 mg/m^2^	[95]
I		CLL/AML(20)	Day1, 8, and 15 of 28-day cycle	13 mg/m^2^	[98]
I	Gemcitabine	Solid tumor(33)	Days 1, 8, and 15 of a 28 day cycle	12 mg/m^2^	[101]
I		Solid tumors(26)	Days 1,3, and 5 of a 28-day cycle	9 mg/m^2^	[100]
II		Renal cell carcinoma(42)	days 1, 8, and 15 of a 28-day cycle	13 mg/m^2^. OR 7%.	[96]
I-II		MDS(3)/AML(9)	Day 1 and 5 of a 21-day cycle	18 mg/m^2 ^(CR .6%, SD 46%, POD 30.7%, NA 7.6%).	[99]
II		SCLC(3)/NSCLC(16)	Day 1 and 7 of a 21 day cycle	SD52%, POD 48%.	[97]

Patients with refractory renal cell cancer were enrolled on a multi-institutional, single-arm, phase II study. Patients received depsipeptide at 13 mg/m2 intravenously over 4 hours on days 1, 8, and 15 of a 28-day cycle with disease reevaluation performed every 8 weeks. The most common serious toxicities were fatigue, nausea, vomiting, and anemia. Two patients developed a prolonged QT interval, one patient each developed grade 3 atrial fibrillation and tachycardia, and there was 1 sudden death. Two patients experienced an objective response for an overall response rate (ORR) of 7% (95% CI, 0.8%-23%). Depsipeptide at this dose and schedule was concluded to have insufficient activity for further investigation in this patient population [96].

Clinical trial in lung cancer exhibited minimal clinical efficacy. Nineteen patients with lung cancer refractory to standard therapy received 4-h depsipeptide infusions (17.8 mg/m^2^) on days 1 and 7 of a 21-day cycle. Each full course of therapy consisted of two identical 21-day cycles. Nineteen patients were evaluated for toxicity assessment; 18 were evaluated for treatment response. Myelosuppression was dose limiting in one individual. No significant cardiac toxicities were observed. Maximum steady-state plasma depsipeptide concentrations ranged from 384 to 1114 ng/mL. No objective responses were observed. Transient SD was noted in nine patients. It may warrant further evaluation of this HDAC inhibitor in combination with novel-targeted agents in lung cancer patients [97].

Chronic lymphocytic leukemia (CLL) and acute myeloid leukemia (AML) cells can be induced by depsipeptide into apoptosis in vitro. Clinical trial was done in ten patients with CLL and 10 patients with AML who were treated with 13 mg/m^2 ^depsipeptide intravenously on days 1, 8, and 15. Neither life-threatening toxicities nor cardiac toxicities were noted, although the majority of patients experienced progressive fatigue, nausea, and other constitutional symptoms that prevented repeated dosing. Depsipeptide effectively inhibits HDAC in vivo in patients with CLL and AML. Several patients had evidence of anti-tumor activity following treatment, but no PRs or complete responses (CRs) were noted. HDAC inhibition and histone acetylation increases of at least 100% were noted. Its use in the current schedule of administration is limited mainly by progressive constitutional symptoms [98]. Another study of depsipeptide was done in patients with myelodysplastic syndrome (MDS) or AML at a dose of 18 mg/m2 intravenously on days 1 and 5 every 3 weeks. Twelve patients (nine with AML, three with MDS) received one to five cycles of depsipeptide. The most common grade 3/4 toxicities were febrile neutropenia/infection (five patients), neutropenia/thrombocytopenia (nine patients), nausea (nine patients), and asymptomatic hypophosphatemia (three patients). No clinically significant cardiac toxicity was observed. One of 11 assessed patients achieved CRs, six in SDs, and four in PODs. The results showed that depsipeptide therapy can be administered with acceptable short-term toxicity. Depsipeptide monotherapy however appears to have limited clinical activity in unselected AML/MDS patients [99].

Another phase I trial of depsipeptide was done following a new schedule. It was administered on days 1, 3 and 5 to a group of twenty six patients with radioactive iodine (RAI)-refractory thyroid cancer. No grade 4 toxicities were observed. Eleven patients had SDs for a median of 28 weeks. Four patients have undergone follow up RAI scans; none had increased RAI uptake. The MTD was reached on this new schedule. This protocol is open exclusively for patients with RAI-refractory thyroid cancer [100].

The combination of depsipeptide and gemcitabine was evaluated in patients with advanced solid tumors. Depsipeptide was administered as a 4 hour infusion followed by gemcitabine over 30 minutes on days 1, 8, and 15 of a 28 day cycle. Thirty-three patients (9 pancreatic, 8 breast, 7 NSCLC, 3 ovarian, 6 other) have received 104+ cycles (median 2, range 1 - 8). Nonhematologic toxicities have been mild to moderate. These consisted primarily of nausea, vomiting, and fatigue. One patient with ovarian cancer experienced a minor response (29%) and 12 patients experienced SDs after ≥ 4 cycles. The phase II dose (depsipeptide 12 mg/m^2 ^and gemcitabine 800 mg/m^2 ^every other week) is being expanded to further assess the safety and activity of the regimen [101].

### Panobinostat (LBH589)

LBH589, a novel hydroxamate analog HDAC inhibitor, has been shown to induce acetylation of histone H3 and H4, increase p21 levels, disrupt the chaperone function of hsp90, and induce cell-cycle G_1 _phase accumulation and apoptosis of K562 cells and acute leukemia MV4-11 cells [102]. The anti-tumor effect by LBH589 was also demonstrated in multiple myeloma, NSCLC as well as castrate-resistant prostate cancer cell lines [30,103-107].

The first clinical trial was done in the patients with hematological malignancy. LBH589 was administered intravenously as a 30-minute infusion on days 1 to 7 of a 21-day cycle (Table 3). Fifteen patients with AML, acute lymphocytic leukemia (ALL), or MDS were treated with LBH589 at the following dose levels (mg/m^2^): 4.8 to 14. The DLTs (grade 3 QTcF prolongation) were observed in four at 14.0 mg/m^2^. QTcF prolongation was asymptomatic and reversed on LBH589 discontinuation. Other potentially LBH589-related toxicities included nausea (40%), diarrhea (33%), vomiting (33%), hypokalemia (27%), loss of appetite (13%), and thrombocytopenia (13%). In 8 of 11 patients with peripheral blasts, transient blast cell reductions occurred with a rebound following the 7-day treatment period. H3 and H2B acetylation increase was significant in B-cells and blasts. Intravenous administration of LBH589 was well tolerated at doses <11.5 mg/m^2 ^with consistent antileukemic and biological effects [108].

**Table 3:** Clinical studies of panobinostat (LBH589)

Phase	Disease (pt. No.)	Schedule	Recommended dose & response	Reference
I	Relapsed or refractory AML (15), MDS (1) and ALL(1).	Day 1 to 7 of a 21-day cycle	11.5 mg/m^2^	[108]
I	Cutaneous T-cell lymphoma(9)	Monday, Wednesday and Friday of each week on a 28-day cycle	20 mg a day, CR 22.2%, PR 44.4%, SD 11.1%, POD 22.2%	[105]
I	Castration-resistant prostate cancer (16)	Arm I: 20 mg on 1,3 and 5 for 2 weeks on a 28-day cycle; Arm II: 15 mg on 1,3 and 5 for 2 weeks on a 28-day cycle with docetaxel and prednisone	Arm I: POD 100%; arm II: PR 37.5%	[112]
II	Advanced CTCL(stage IB-IVA) Group 1 previously treated with bexarotene(25); group 2 bexarotene naïve(15)	Days 1,3, and 5 weekly until disease progression or intolerance	Group 1: PR12%, SD16%, PD12%;other patients and most patients in groups have had less than 2 months of follow-up.	[109]

The patients with CTCL (stage IB-IVA) were enrolled in an open-label clinical trial study to measure the safety and toxicity of LBH589. Patients included Mycosis fungoides (MF) and Sezary syndrome (SS), who have failed ≥2 prior systemic therapies. Patients were assigned to two different groups: Group 1 previously treated with oral bexarotene or Group 2 without bexarotene. Panobinostat (20 mg) was administered orally on days 1, 3, and 5 weekly until disease progression or intolerance. Most common (>15%) side effects include diarrhea, thrombocytopenia, fatigue, asthenia, hypertriglyceridaemia, dysgeusia, nausea and pruritus. Intensive ECG monitoring for QTc prolongation was performed. Among 1578 ECGs analyzed, there has been no QTc >500 ms, one QTc >480 ms, and one QTc >60 ms increased from baseline. Best overall response is PR for 3 patients, SD for 4 patients. Preliminary safety data suggest that panobinostat is generally well tolerated [109]. Microarray data showed that panobinostat induced distinct gene expression profiles over time following treatment, with the majority of genes being repressed. Panobinostat regulated twenty-three common genes in all patients tested. A unique set of genes that can mediate biological responses such as apoptosis, immune regulation, and angiogenesis were commonly regulated in response to panobinostat. These genes are strong candidates for the future assessment of their functional role in mediating the anti-tumor responses of panobinostat [105].

HDAC inhibitors can block androgen receptor -mediated transcriptional activation of many genes and thus may result in possible benefit in treating Castration-resistant prostate cancer [110]. Docetaxel is first line therapy for patient with castration-resistant prostate cancer [111]. Phase I Clinical study with oral panobinostat alone or in combination with docetaxel in castration-resistant prostate cancer showed that oral panobinostat with and without docetaxel is feasible and a drug-drug interaction is not apparent. 16 patients were enrolled in this study. DLTs include dyspnea and neutropenia. Three patients achieved a PR as best response. Two of these three patients elected to hold treatment due to fatigue. All evaluable patients at the 20 mg single agent dose (7/7) demonstrated accumulation of acetylated histones in monocytes [112].

### MGCD0103

MGCD0103 is a novel isotype-selective inhibitor of human HDACs with the potential to regulate aberrant gene expression and restore normal growth control in malignancies. A phase I trial of MGCD0103, given as a three-times-per-week oral dose for 2 of every 3 weeks, was performed in patients with advanced solid tumors (Table 4). DLTs consisting of fatigue, nausea, vomiting, anorexia, and dehydration were observed in three (27%) of 11 and two (67%) of three patients treated at the 45 and 56 mg/m^2^/d dose levels, respectively. SD was observed after four or more cycles of treatments in five (16%) of 32 patients assessable for efficacy. PK analyses demonstrated inter-patient variability which was improved by co-administration with low pH beverages. Elimination half-life ranged from 6.7 to 12.2 hours, and no accumulation was observed with repeated dosing. Pharmacodynamic (PD) evaluations confirmed inhibition of HDAC activity and induction of acetylation of H3 histones in peripheral WBCs from patients by MGCD0103. The recommended phase II dose was 45 mg/m^2^/day. At doses evaluated, MGCD0103 appears to be tolerable and exhibits favorable PK and PD profiles with evidence of target inhibition in surrogate tissues [113].

**Table 4:** Clinical studies of MGCD0103

Phase	Other agent	Disease (pt. No.)	Schedule	Recommended dose & response	Reference
I		Advanced solid tumor(38)	Three times per week for 2 of every three weeks	45 mg/m^2^/d	[113]
I		Relapsed or refractory AML (22), MDS (5), ALL(1) and CML (1)	Three times weekly without interruption	60 mg/m^2^/d	[114]
I/	Gemcitabine	Solid tumor(24/I and 4/II)	Three times weekly for MGCD0103 and weeklyX3 for Gemcitabine in 28-days cycle	90 mg/d and PR: 40% in 2 out of 5 pancreatic carcinoma.	[115]
II		Relapsed or refractory NHL(33 of DLBCL and 17 of FL)	Three times weekly without interruption	Started 110 mg, then decreased to 85 mg. RR for DLBCL 23.5% and PR for FL 10%.	[116]
II		Relapsed or refractory HL(33)	Three times weekly in 28 days cycle	85 mg or 110 mg. OR 38%.	[117]

MGCD0103 was also studied in patients with leukemia and MDS. Patients were treated with 3 times weekly schedule without interruption in this phase I study. The MTD was 60 mg/m2, with DLTs of fatigue, nausea, vomiting, and diarrhea observed at higher doses. Three patients achieved a complete bone marrow response. PK analyses indicated absorption of MGCD0103 within 1 hour and an elimination half-life in plasma of 9 (+/- 2) hours. In summary, MGCD0103 was well tolerated and had antileukemia activity [114].

MGCD0103 combined with gemcitabine had demonstrated more effective anti-tumor activity than alone in pre-clinical studies. Phase I/II study with MGCD0103 alone or combination with gemcitabine were done in patients with solid tumors recently. Phase I part of the trial studied adults with refractory solid tumors. Phase II part of the trial was limited to gemcitabine naive patients with locally advanced or metastatic pancreatic cancer. Patients received MGCD0103 (3 times a week) in 28-day cycles at sequential ascending doses using a 3+3 design targeting a DLT rate of <33%. Gemcitabine was administered at 1,000 mg/m^2^, weekly × 3 per cycle. DLTs included fatigue, vomiting and abdominal pain as well as thrombocytopenia and anemia. Inhibition of HDAC activity was observed in patients' PBMCs. The MTD and recommended phase II dose was 90 mg. Among 14 response-evaluable phase I patients, there were 2 PRs out of 5 pancreatic carcinoma patients and 2 PRs in a patient with nasopharyngeal cancer and a patient with cutaneous T- cell lymphoma. Two patients were observed with SD after receiving >2 cycles (1 lung and 1 pancreatic). The combination may have clinical activity in patients with solid tumors in general and pancreatic cancer in particular. Phase II at the dose of 90 mg of MGCD0103 is ongoing in patients with pancreatic cancer [115].

Open-label, phase II trial in adults with relapsed or refractory diffuse large B-cell lymphoma (DLBCL) or follicular lymphoma (FL) also demonstrated significant anti-cancer activity with manageable side effect profile. Fifty patients received treatment; including 33 DLBCL and 17 FL. Of 17 DLBCL patients with tumor reassessed by CT, most had tumor reduction, including 1 CR & 3 PRs, with progression free survival (PFS) for responders ranging from 168 to >336 days. Five DLBCL patients with stable disease had PFS ranging from 112 to >336 days. One of 10 FL patients achieved PR. The most common toxicities of grade ≥3 were fatigue (14%), neutropenia (12%), thrombocytopenia (10%), and anemia (6%) [116].

Since Hodgkin's lymphoma (HL) patients with relapsed or refractory disease have poor prognosis, an open-label, phase II trial in adults with relapsed/refractory HL was conducted. Patients received MGCD0103 at 110 or 85 mg 3 times per week in 4-week cycles. Among 23 patients in the 110 mg cohort, 21 were evaluated, of whom 2 (10%) had CRs and 6 (29%) had PRs for an ORR of 38%. The 2 patients with CRs had progression free survival lasting >270 and >420 days, respectively, with both responses ongoing. One additional patient (5%) had SD >6 cycles. Among 10 patients in the 85 mg cohort, 5 were evaluated for efficacy, all of whom had tumor reductions of ≥30%; including 1 PR and 2 SDs. MGCD0103 demonstrated significant anti-tumor activity in relapsed/refractory HL [117].

### Belinostat (PXD101)

The activity of belinostat was investigated in many cell lines, which include hepatocellular carcinoma, human cancer, chronic lymphocytic leukemia, prostate cancer, bladder cancer, head and neck squamous carcinomas and ovarian cancer cells in preclinical studies [118-126].

In a phase I clinical trial, forty-six patients with advanced refractory solid tumors received belinostat at one of six dose levels (150-1200 mg/m^2^/d). DLTs were fatigue, diarrhea, atrial fibrillation; and grade 2 nausea/vomiting leading to inability to complete a full 5-day cycle. The MTD was 1000 mg/m2/d. The intermediate elimination half-life was 0.3 to 1.3 h and was independent of dose. SD was observed in a total of 18 (39%) patients, including 15 treated for more than 4 cycles. Of the 24 patients treated at the MTD, 50% achieved SD. Belinostat exhibits dose-dependent pharmacodynamic effects, and has promising anti-tumor activity (Table 5) [127].

**Table 5:** Clinical studies of belinostat (PXD101)

Phase	Other agent	Disease (pt. No.)	Schedule	Recommended dose & response	Reference
I		Advanced hematological neoplasms(16)	Day 1 to 5 of a 21-day cycle	1000 mg/m^2^/d	[128]
I	AZA	Advanced myeloid neoplasms(230	Days 1-5 of a 28 day cycle	1000 mg/m^2^	[129]
I		Advanced refractory solid tumors(46)	Days 1-5 of a 21 day cycle	1000 mg/m^2^ SD 39%	[127]
II		relapsed malignant pleural mesothelioma(13)	Days 1-5 of a 21 day cycle	Belinostat is not active as monotherapy against recurrent malignant pleural mesothelioma	[122]
II		Platinum resistant epithelial ovarian tumors(EOC,18) and micropapillary/borderline ovarian tumors(LMP,12)	Days 1-5 of a 21 day cycle with 1000 mg/m2	EOC: SD 50%, PD25% N/E 25%; LMP: SD 75%, PR8.3%, N/E 16.6%	[130]

Sixteen patients with advanced hematological neoplasms received belinostat in another clinical trial at one of three dose levels: 600 mg/m^2^/d, 900 mg/m^2^/d and 1000 mg/m^2^/d. The most common treatment-related adverse events were nausea, vomiting, fatigue and flushing. No grade 3 or 4 hematological toxicity compared with baseline occurred except one case of grade 3 lymphopenia. There were two grade 4 renal failure. Both events occurred in patients with multiple myeloma. No cardiac events were noted. No CRs or PRs were noted in these heavily pre-treated patients. However, five patients, including two patients with diffuse large-cell lymphoma achieved SD after two to nine treatment cycles. Intravenous belinostat at 600, 900 and 1000 mg/m^2^/d was well tolerated. 1000 mg/m^2^/d on days 1-5 in a 21-d cycle was recommended for phase II studies in patients with hematological neoplasia [128].

Simultaneously targeting two epigenetic pathways using belinostat and the DNA hypomethylating agent azacitidine (AZA) may lead to an additive or synergistic effect in patients with advanced myeloid neoplasms. AZA, 75 mg/m^2^/d, was given subcutaneously on days 1-5 followed by escalating doses of belinostat given intravenously over 30 minutes on the same days in a 28 day cycle. Twenty one patients received at least 1 cycle and are evaluated for response: 2 CRs, 1 PR and 4 with hematologic improvement. Median time to response was 2 cycles. Increased platelets at 4 weeks were observed in one-third of patients at all dose levels studied. The combination of belinostat with AZA is feasible. A randomized study was suggested to further investigate the relative contribution of belinostat to clinical efficacy [129].

Patients with low malignant potential (LMP) ovarian tumors represent an understudied population whose tumors are intrinsically resistant to radiation and chemotherapy. Patients with platinum resistant epithelial ovarian cancer (EOC) have low response rates to conventional chemotherapy too. Belinostat demonstrates anti-tumor activity in ovarian cancer animal models. Two patient populations, metastatic or recurrent platinum resistant (< 6 mo) EOC and LMP ovarian tumors, were enrolled to assess the activity of belinostat. Belinostat 1,000 mg/m^2^/day was administered intravenously on days 1-5 of a 21 day cycle. The most frequent grade 3 adverse events were bowel obstruction, thrombosis, dyspnea, fatigue, lymphopenia, elevated ALP and nausea. Eighteen patients with EOC received a total of 50 cycles of treatment. 9 patients had SDs, 6 PODs, 3 are non evaluable and 2 remained on study. 12 patients with LMP tumors received 68 cycles of treatment. 1 patient had a PR, 9 SDs, and 2 are non evaluable. Belinostat showed promising activity in LMP ovarian tumors [130].

Thirteen patients with advanced mesothelioma with progression on one prior chemotherapy regimen have been recruited to a phase II study using belinostat. SD was seen in two patients. No objective responses were noted. One patient died as a consequence of cardiac arrhythmia. It was concluded that belinostat is not active as monotherapy against recurrent malignant pleural mesothelioma. Evaluation of combination strategies was suggested for further development of this novel agent in mesothelioma [122].

### Valproic acid

Valproic acid (VPA) can induce in vitro differentiation of primary AML blasts in vitro. Seventy five patients with AML/MDS were enrolled in a clinical trial (Table 6). Of these, sixty six were started on VPA monotherapy, with later addition of all trans-retinoic acid (ATRA) in patients who did not respond or relapsed. Median treatment duration was 4 months for VPA and 2 months for ATRA. Hematological improvement was observed in 18 patients (24%). Median response duration was 4 months. ATRA exerted no additional effect in patients receiving the combination. However, of ten VPA responders who relapsed, four achieved a second response after addition of ATRA. Response rates were strongly dependent on disease type according to WHO classification. There was a response rate of 52% in MDS patients with a normal blast count. The response rate was 6% in refractory anemia with excess blasts (I + II), 16% in AML, and 0% in chronic myelomonocytic leukemia [131]. Another clinical study in similar patient population showed that treatment with VPA/ATRA combination results in transient disease control in a subset of patients with AML that has evolved from a myeloproliferative disorder but not in patients with a primary or MDS-related AML [132,133]. In another study of 54 patients with AML/MDS, a fixed dose of decitabine (15 mg/m^2 ^by IV daily for 10 days) was administered concomitantly with escalating doses of VPA orally for 10 days. A 50 mg/kg daily dose of VPA was found to be safe. Twelve (22%) patients had objective response, including 10 (19%) CRs, and 2 (3%) CRs with incomplete platelet recovery. In summary, this combination of epigenetic therapy in leukemia appears to be safe and active, and was associated with transient reversal of aberrant epigenetic marks [134]. However, in a separate phase I study, encephalopathy was seen in AML patients treated with VPA plus Low-dose decitabine (20 mg/m^2^/d for 10 days) [135].

**Table 6:** Clinical studies of valproic acid

Phase	Other agent	Disease (pt. No.)	Schedule	Recommended dose & response	Reference
I	ATRA(80 mg/m2)	AML (58).	Twice a day	VPA serum concentration to 50-100 ug/ml	[131]
I		Cervical cancer(12)	Once a day	20-40 mg/kg	[137]
I	ATRA(45 mg/m2)	AML(26)	Once a day	5-10 mg/kg	[132]
I	Decitabine (5 mg/m2)	NSCLC(8)	5-aza-CdR for 10 days in combination with VA on days 5-21 of a 28-day cycle.	15 mg/kg/d	[140]
I		Refractory advanced cancer(26)	Daily for 5 days in a 21-day cycle	60 mg/kg/day	[138]
I	Epirubicin	Solid tumor(44)	Daily for three days then followed by epirubicin in 21 day cycle	VPA 140 mg/kg/d Epirubincin 100 mg/m2	[139]
I	AZA and ATRA	AML(49) and MDS(4)		VPA 75 mg/kg	[136]
I and II	Decitabine(15 mg/m2)	AML(54)	Once a day	50 mg/kg, 22% objective response	[134]

Soriano *et al*. conducted a phase I/II study of the combination of AZA, VPA, and ATRA in patients with AML or high-risk MDS. AZA was administered at a fixed dose of 75 mg/m^2 ^daily for 7 days. VPA was dose-escalated and given orally daily for 7 days concomitantly. ATRA was given at 45 mg/m^2 ^orally daily for 5 days, starting on day 3. A total of 53 patients were treated. The MTD dose of VPA in this combination was 50 mg/kg daily for 7 days. DLT was reversible neurotoxicity. The ORR was 42%. Median remission duration was 26 weeks. In conclusion, the combination studied is safe and has significant clinical activity [136].

The activity of VPA was also evaluated on solid tumors. Twelve patients with cervical cancer were enrolled for phase I trial in 2005. The patients were treated with VPA after a baseline tumor biopsy and blood sampling at the following dose levels (four patients each): 20 mg/kg; 30 mg/kg, or 40 mg/kg for 5 days via oral route. At day 6, tumor and blood sampling were repeated and the study protocol ended. Blood levels of VPA were measured at day 6 once the steady-state was reached. Mean daily dose for all patients was 1890 mg. Depressed level of consciousness of grade 2 was registered in nine patients. Serum levels of VPA ranged from 73.6-170.49 ug/mL. Tumor deacetylase activity decreased in eight patients with a statistically significant difference between pre and post-treatment values of HDAC activity (p < 0.0264). No correlation between tumor hyperacetylation with serum levels of valproic acid was found [137]. Another phase I study in Twenty-six pre-treated patients with progressing solid tumors also showed that neurocognitive impairment dominated the toxicity profile, with grade 3 or 4 neurological side effects occurring in 8 out of 26 patients. No grade 3 or 4 hematological toxicity was observed. The MTD of infusion VPA was 60 mg kg/day. Further investigations are warranted to evaluate the effect of VPA alone and in combination with other cytotoxic drugs [138].

In another phase I study, a sequence-specific combination of VPA and epirubicin in solid tumor malignancies was done. Patients were treated with increasing doses of VPA for three days followed by epirubicin in 3-week cycles. The study evaluated PK and PD end points, toxicities, and tumor response. DLTs were similar to that seen with single agent VPA. No exacerbation of epirubicin-related toxicities was observed. The MTD and recommended phase II dose was VPA 140 mg/kg/d for 48 hours followed by epirubicin 100 mg/m^2^. PRs were seen across different tumor types in nine patients (22%), and SDs were seen in 16 patients (39%). Anti-tumor activity was observed in heavily pretreated patients and historically anthracycline-resistant tumors [139]. In another phase I study in patient with metastatic NSCLC, combination of decitabine at dose 5 mg/m^2 ^for 10 days with VPA at 10 mg/kg/d on days 5-21 of a 28 day cycle was not well tolerated. Further study of decitabine at a five day schedule in combination with HDAC inhibitors is ongoing [139,140].

A phase II study of hydralazine and VPA in treating patients with advanced solid tumors revealed clinical benefit. Primary tumor included cervix (3), breast (3), lung (1), testis (1), and ovarian (7) carcinomas. Clinical benefit was observed in 12 (80%) patients: four PRs, and eight SDs. The most significant toxicity was hematological [141].

## Conclusions

Targeted therapy is widely used nowadays for cancer treatment. The targeting agents include inhibitors of tyrosine kinases, angiogenesis, mTOR, and epigenetic pathways, to name a few [142-145]. Besides vorinostat, there are more than 8 other HDAC inhibitors undergoing active clinical investigation. It is noteworthy that ITF2357 showed significant anti-HL activity. Panobinostat showed consistent anti-leukemic effects. Belinostat appears to be promising for treating LMP ovarian tumor. The combination of AZA, VPA, and ATRA has significant clinical activity in leukemia and MDS. Epigenetic agents in combination regimens for cancer therapy are being actively studied.

## Abbreviations

AML: Acute myeloid leukemia; ALL: Acute lymphocyte leukemia; AR: Androgen receptor; ATRA: All-trans retinoic acid; CLL: Chronic lymphocyte leukemia; CML-BC: Chronic myeloid leukemia blast crisis; CR: Complete response; CTCL: Cutaneous T-cell lymphoma; DLBCL: Diffuse large B-cell lymphoma; DLTs: Dose-limiting toxicities; DNMT: DNA methyltransferase; EOC: Epithelial ovarian cancer; FL: Follicular lymphoma; HDACs: Histone deacetylases; HL: Hodgkin lymphoma; LMP: Low malignant potential; MF: Mycosis fungoides; MDS: Myelodysplastic syndrome; MTD: Maximum tolerated dose; NHL: Non Hodgkin lymphoma; NSCLC: Non small cell lung carcinoma; ORR: Overall response rate; PBMCs: Peripheral blood mononuclear cells; PD: Pharmacodynamic: Progression free survival; PK: Pharmacokinetics; POD: Progression of disease; PR: Partial response; RAI: Radioactive iodine; SAHA: Suberoyl anilide hydroxamic acid; SD: Stabilization of disease; SS: Sezary syndrome.

## Competing interests

The authors declare that they have no competing interests.

## Authors' contributions

JT and DL are involved in concept design. All authors participated in data collection, drafting and critically revising the manuscript.
